# Fostering Innovation in the Treatment of Chronic Polymicrobial Cystic Fibrosis-Associated Infections Exploring Aspartic Acid and Succinic Acid as Ciprofloxacin Adjuvants

**DOI:** 10.3389/fcimb.2020.00441

**Published:** 2020-08-27

**Authors:** Eduarda Silva, Rosana Monteiro, Tânia Grainha, Diana Alves, Maria Olivia Pereira, Ana Margarida Sousa

**Affiliations:** CEB - Centre of Biological Engineering, LIBRO – Laboratório de Investigação em Biofilmes Rosário Oliveira, University of Minho, Braga, Portugal

**Keywords:** *Pseudomonas aeruginosa*, cystic fibrosis, biofilms, adjuvants, ciprofloxacin, aspartic acid, succinic acid

## Abstract

Cystic fibrosis (CF) disease provokes the accumulation of thick and viscous sputum in the lungs, favoring the development of chronic and polymicrobial infections. *Pseudomonas aeruginosa* is the main bacterium responsible for these chronic infections, and much of the difficulty involved in eradicating it is due to biofilm formation. However, this could be mitigated using adjuvant compounds that help or potentiate the antibiotic action. Therefore, the main goal of this study was to search for substances that function as adjuvants and also as biofilm-controlling compounds, preventing or dismantling *P. aeruginosa* biofilms formed in an *in vitro* CF airway environment. Dual combinations of compounds with subinhibitory (1 and 2 mg/L) and inhibitory concentrations (4 mg/L) of ciprofloxacin were tested to inhibit the bacterial growth and biofilm formation (prophylactic approach) and to eradicate 24-h-old *P. aeruginosa* populations, including planktonic cells and biofilms (treatment approach). Our results revealed that aspartic acid (Asp) and succinic acid (Suc) restored ciprofloxacin action against *P. aeruginosa*. Suc combined with 2 mg/L of ciprofloxacin (Suc-Cip) was able to eradicate bacteria, and Asp combined with 4 mg/L of ciprofloxacin (Asp–Cip) seemed to eradicate the whole 24-h-old populations, including planktonic cells and biofilms. Based on biomass depletion data, we noted that Asp induced cell death and Suc seemed somehow to block or reduce the expression of ciprofloxacin resistance. As far as we know, this kind of action had not been reported up till now. The presence of *Staphylococcus aureus* and *Burkholderia cenocepacia* did not affect the efficacy of the Asp–Cip and Suc–Cip therapies against *P. aeruginosa* and, also important, *P. aeruginosa* depletion from polymicrobial communities did not create a window of opportunity for these species to thrive. Rather the contrary, Asp and Suc also improved ciprofloxacin action against *B. cenocepacia*. Further studies on the cytotoxicity using lung epithelial cells indicated toxicity of Suc–Cip caused by the Suc. In conclusion, we provided evidences that Asp and Suc could be potential ciprofloxacin adjuvants to eradicate *P. aeruginosa* living within polymicrobial communities. Asp–Cip and Suc–Cip could be promising therapeutic options to cope with CF treatment failures.

## Introduction

Cystic fibrosis (CF) patients suffer from accumulation of thick and viscous sputum in the airway tract that predisposes them to the development of airway chronic infections (Ciofu et al., 2014). A complex community of microbes encompassing bacteria, fungi, and even viruses can colonize the lungs of CF patients and establish chronic infections responsible for the increased mortality rate of these patients (Dickson et al., 2013; Magalhães et al., 2016a). Within the CF microbiome, *Pseudomonas aeruginosa* is considered the most relevant pathogenic agent responsible for the development of chronic infections, decreased quality of life, long hospitalizations, and ultimately the number of deaths (Bittar et al., 2008; Bhagirath et al., 2016). Once *P. aeruginosa* enters in the CF lungs, it is hard to be eradicated, even using aggressive and long antibiotic treatments. The remarkable switch of *P. aeruginosa* between an acute and CF-adapted chronic phenotype exhibiting antibiotic resistance, biofilm formation ability, overproduction of alginate (mucoid phenotype), slow growth rate (small colony variants, SCV), and loss of motility seems to be the key for its persistence in CF lungs (Sousa and Pereira, 2014; Sousa et al., 2018). Moreover, it is becoming increasingly clear that interspecies interactions can influence the pathogenesis of *P. aeruginosa*, also contributing for its persistence and worsening of the patient's condition (Magalhães et al., 2016b; O'Brien and Fothergill, 2017). For instance, CF patients coinfected with *P. aeruginosa* and *Staphylococcus aureus* have more severe effects on lung function and poorer clinical prognosis than the patients with mono-infections (Hubert et al., 2013). The cohabitation with *S. aureus* hampers the host immune response against *P. aeruginosa* and increases *P. aeruginosa* virulence and tolerance to antibiotics (Beaudoin et al., 2017; Hotterbeekx et al., 2017; Limoli et al., 2017). Not so much frequent but still concerning is the co-isolation of *P. aeruginosa* with *Burkholderia cenocepacia* that is typically associated with severe infections because of the increase biofilm formation and host inflammatory response (Coutinho et al., 2011; Bragonzi et al., 2012).

Numerous antimicrobial strategies have been used varying in the route of antibiotic administration (systemic, oral, inhaled antibiotics, or route combination), classes of antibiotics and treatment duration in attempt to eradicate *P. aeruginosa* chronic infections (Waters and Smyth, 2015; Emiralioglu et al., 2016). However, these therapies are often unsuccessful, mostly due to biofilms that are microbial communities encased in a self-produced matrix that provide to the resident organisms augmented tolerance to antibiotics (Lopez et al., 2010). Their impressive high level of antimicrobial tolerance results from the combination of several mechanisms, including restricted penetration of antibiotics through the matrix, slow growth of bacteria within biofilms due to nutritional constraints and restricted oxygen penetration, quorum-sensing, expression of biofilm-specific genes, and the presence of persister cells (Mah, 2012; Jorge et al., 2019).

The lack of new antibiotics on the horizon forces the community to seek alternative therapeutic strategies in order to restore the action of the in-use antibiotics. A promising approach is the use of “helper compounds” or adjuvants that enhance the activity of antibiotics. By definition, antibiotic adjuvants are compounds with little or no antibiotic activity, but when combined or coadministered with an antibiotic, they block the main resistance mechanisms or potentiate the antibiotic action (Kalan and Wright, 2011; Brown, 2015). Adjuvant therapies typically include antibiotic combinations, long used in CF therapeutics (e.g., ciprofloxacin and colistin) and synergy between antibiotics and non-antibiotics (e.g., ß-lactamase inhibitors, efflux pump inhibitors, outer-membrane permeabilizers) (Kalan and Wright, 2011; Döring et al., 2012). However, antibiotic adjuvants can also include compounds that alter the physiological state of antibiotic tolerant cells, such as those in biofilms (Kalan and Wright, 2011). Compounds that act on the prevention of biofilm formation, disruption of biofilm integrity, or dispersal to planktonic state (a more susceptible state) might have great potential to function as antibiotic adjuvants.

Several natural, synthetic, or biological anti-biofilm approaches have been investigated including nutrient-induced dispersals (e.g., succinate, glutamate, glucose, nitrite oxide, D-amino acids), matrix disruptive enzymes (e.g., DNase I, Dispersin B, alginate lyase, glutathione), iron chelators (e.g., EDTA, lactoferrin), quorum-sensing inhibitors, bacteriophages, and altered pH (Sauer et al., 2004; Banin et al., 2006; Pires et al., 2011; Lebeaux et al., 2014a; Brackman and Coenye, 2015; Chambers et al., 2017; Das et al., 2017; Aliashkevich et al., 2018). Despite that some of these approaches having demonstrated some effect against *P. aeruginosa* biofilms, they were not tested on biofilms formed in conditions that mimic the CF airway environment. Airway CF biofilms are genetic, proteomic, and physiologically different from the surface-attached biofilms that most of the studies use in their investigations. Instead of the direct surface colonization, bacteria in the CF lungs preferentially form aggregates and biofilms within the sputum and not on the surface epithelium in the bronchi and non-respiratory bronchioles (Singh et al., 2000; Worlitzsch et al., 2002; Bjarnsholt et al., 2009). Therefore, the main goal of this study was to find compounds that can act as antibiotic adjuvants augmenting ciprofloxacin action against polymicrobial biofilms containing *P. aeruginosa* formed in an *in vitro* CF airway environment. To mimic the airway CF biofilms, we used a previously established artificial sputum medium (ASM) to closely simulate the physicochemical conditions of CF lungs and promote the formation of free-floating biofilms within ASM as demonstrated by Sriramulu et al. (2005). The dual therapies of compounds and ciprofloxacin were applied before bacterial growth in ASM, prophylaxis approach, to prevent or minimize bacterial growth and biofilm formation, and after 24 h of bacterial growth, the treatment approach, to dismantle biofilms and eradicate planktonic and sessile cells. In this study, we selected ciprofloxacin because it is one of the most prescribed antibiotics to treat *P. aeruginosa* infections, including those developed in CF lungs (Döring et al., 2012; Remmington et al., 2016). The compounds selected to act as adjuvants were previously described as broad-spectrum biofilm-controlling agents that did not target essential biological functions, but they had an extensive action on biofilms. They included (i) succinic acid as a carbon source that promoted biofilm-cell dispersion to planktonic state (Sommerfeld Ross and Fiegel, 2012); (ii) aspartic acid and glutathione as disrupting agents of biofilm integrity (Klare et al., 2016; Bahamondez-Canas and Smyth, 2018); (iii) galactose as anti-adhesion agent inhibiting biofilm formation (Hauber et al., 2008); and (iv) acylases I as quorum sensing inhibitor (Xu et al., 2003).

## Materials and Methods

### Bacterial Strains and Culture Conditions

*P. aeruginosa* PAO1 and a clinical isolated PAI were used throughout this study. The rationale behind the selection of these strains was based on their distinct susceptibility to ciprofloxacin determined in this study. PAO1 is a laboratory and low-virulence strain, and PAI is a respiratory clinical isolate kindly provided by the Hospital of Braga (Braga, Portugal) (Pires et al., 2011). One CF clinical isolate of *S. aureus* and *B. cenocepacia* was used to evaluate the influence of the most relevant non-pseudomonal species resident in CF lungs on the efficacy of the dual therapies. Bacteria were routinely cultured on Tryptic Soy Broth (TSB, Liofilchem) or Tryptic Soy Agar (TSA, Liofilchem) at 37°C. All strains were preserved in criovials (Nalgene) at −80 ± 2°C to minimize putative adaptation to the laboratory environment. Prior to each experiment, bacterial cells were grown on TSA plates overnight at 37°C.

### Determination of Minimum Inhibitory Concentration (MIC)

The antibiotic susceptibility of *P. aeruginosa* strains was determined by MIC using the microdilution assay following the recommendations of the Clinical and Laboratory Standards Institute (CLSI, formerly the National Committee for Clinical Laboratory Standards) (CLSI 2007). Overnight cultures of *P. aeruginosa* strains were washed and diluted with Mueller Hinton Broth (MHB) to 5 × 10^5^ CFU/mL and transferred to 96-well plates. Further, bacterial strains were exposed to different concentrations of ciprofloxacin (Fluka) ranging from 0.03 to 16 mg/L at 37°C, 120 rpm, for 18–21 h in air conditions. MIC was determined by the minimum concentration of antibiotic required to inhibit 90% of growth, measuring the optical density at 620 nm on a microtiter plate reader (EZ Read 800 Plus, Biochrom). The clinical breakpoint for ciprofloxacin was 4 mg/L as defined by CLSI (CLSI 2007). MHB alone and bacterial cultures free of ciprofloxacin were used as negative and positive controls, respectively. All tests were performed five times (independent biological assays) with three technical replicates.

### *In vitro* Growth of *P. aeruginosa* in an *in vitro* CF Airway Environment

#### Artificial Sputum Medium Preparation

Artificial sputum medium (ASM) was used to mimic the sputum of CF patients, and it was prepared as described by Sriramulu et al. (2005). Briefly, 5 g/L of mucin from pig stomach (Sigma-Aldrich), 4 g/L of DNA from salmon sperm (Sigma-Aldrich), 5.9 mg/L of diethylenetriaminepentaacetic acid (DTPA, Sigma-Aldrich), 5 g/L of NaCl, 2.2 g/L of KCl, and 5 g/L of casoamino acids (AMESRO) were resuspended in water and the pH adjusted to 7 with Tris base. This ASM was then sterilized in an autoclave at 110°C for 15 min, and after being cooled, 5 mL of egg yolk emulsion (Fluka) was added.

#### Preparation of Stock Solutions of the Compounds

Distilled sterile water was used for water-soluble molecules, including glutathione (Sigma-Aldrich), galactose (Sigma-Aldrich), and succinic acid (VWR Chemicals). Galactose, L-aspartic acid, and acylases I were dissolved in 0.9 M NaCl, 2 M NaOH, and 100 mM potassium phosphate buffer, pH 7, respectively, according to the manufacturer's recommendations (Sigma-Aldrich). Concentrated solutions of the compounds were prepared and applied to ASM cultures to obtain a final concentration of 20 mM of aspartic acid, succinic acid, and galactose, 10 mM of glutathione, and 5 and 15 mg/L of acylases I as described in literature (Xu et al., 2003; Sauer et al., 2004; Klare et al., 2016; Bahamondez-Canas and Smyth, 2018). All stock solutions were freshly prepared before their application on ASM. Three different concentrations of ciprofloxacin, two subinhibitory concentrations and one inhibitory concentration according to the MIC values obtained for the *P. aeruginosa* strains, were combined with the compounds.

#### Anti-pseudomonal Activity of Ciprofloxacin Combined With Compounds

Dual therapies were applied as prophylaxis, before *P. aeruginosa* growth in ASM, and as treatment, after 24 h of bacterial growth in ASM and the formation of biofilms, as demonstrated by Sriramulu et al. (2005). In the prophylactic application, dual therapies were first applied to ASM followed by *P. aeruginosa*. Overnight inocula of each *P. aeruginosa* strain were washed twice in sterile water by centrifugation (9,000 *g*, 5 min) and further serial diluted in sterile water. Two milliliters of ASM was transferred to each well of a 24 well-plate (polystyrene, Orange, USA), and the dual therapies were added. After 30 min, ASM was inoculated on the top with 5 μL of the bacterial cell suspensions, obtaining a final cellular concentration in each well of 1 × 10^7^ CFU/mL. ASM cultures were incubated for 24 h at 37°C aerobically in static culture conditions to resemble the reduced or absent cilia movements in CF lungs (Worlitzsch et al., 2002; Hassett et al., 2009). The content of the wells was then collected aseptically and vigorously shaken to detach cells from possible small aggregates or cells adhered to the mucin. The resulting cell suspensions, corresponding to the whole resident populations in ASM, were plated on TSA to determine the number of culturable cells that survived after the prophylactic application of the dual therapies. In the treatment approach, *P. aeruginosa* was first added to ASM as aforementioned and allowed to grow for 24 h to form biofilms as demonstrated by Sriramulu et al. (2005). After, the dual therapies were added to ASM, containing the 24-h-old planktonic cells and biofilms, and their efficacy evaluated after 24 h by counting the culturable cells that survived. All experiments were performed at least five times.

#### Antimicrobial Effect of the Dual Therapies on Polymicrobial Communities

The prophylactic effectiveness of the dual therapies for eradication of the polymicrobial communities of bacteria from ASM was determined as described above with few modifications. First, ASM was inoculated with bacterial cell suspensions of *S. aureus* and *P. aeruginosa* or with *B. cenocepacia* and *P. aeruginosa*, obtaining a final concentration of 1 × 10^7^ CFU/mL for each bacterial species. ASM cultures were incubated at 37°C for 24 h aerobically and in static culture conditions, and the number of culturable cells that survived determined. To investigate the effectiveness of the treatment approach, ASM was first inoculated with bacterial cell suspensions of *S. aureus* and *P. aeruginosa* or with *B. cenocepacia* and *P. aeruginosa* at a final concentration of 1 × 10^7^ CFU/mL for each species and cultures incubated at 37°C for 24 h aerobically and in static culture conditions to allow mixed biofilm formation. After, the ASM containing mixed planktonic cells and 24-h-old biofilms was exposed to the dual therapies for 24 h. The whole content of ASM, corresponding to the whole resident populations, was then plated on selective solid media to discriminate each species. *P. aeruginosa* was plated on *Pseudomonas* isolation agar (Sigma-Aldrich) or cetrimide (Liofilchem) if it grew with *S. aureus* or *B. cenocepacia*, respectively. *S. aureus* was plated on Mannitol salt agar (Liofilchem) and *B. cenocepacia* was plated on *Burkholderia* selective agar (Oxoid) with 150 000 IU/L of polymyxin B, 5 mg/L of gentamicin, and 100 mg/L of ticarcillin.

#### Cytotoxicity of the Dual Therapies on Lung Epithelial Cells

Cytotoxicity of the most promising dual therapies was evaluated on human lung epithelial A549 cells (ATCC CCL-185). Cells were grown in Dulbecco modified eagle medium (DMEM, Gibco) supplemented with 10% of fetal bovine serum (FBS, Gibco) and 1% antibiotics (ZellShield™, Biochrom) at 37°C, 5% CO_2_. Once having achieved a minimum of 80% confluence, cells were detached, using trypsin, and adjusted to a final concentration of 1 × 10^5^ cells/mL. Then, 100 μL of cell suspension was transferred to each well of a 96-well plate, which was incubated for 24 h at 37°C, 5% CO_2_. After this period, cell culture supernatant was removed and 100 μL of dual therapies prepared in supplemented DMEM was added. Fresh culture media with no compounds were also added as a positive control. The plate was incubated for an additional 24 h at 37°C, 5% CO_2_. Metabolic activity of cells was determined using the MTS [(3-(4,5-dimethylthiazol-2-yl)-5-(3-carboxymethoxyphenyl)-2-(4-sulfophenyl)-2H-tetrazolium)] inner salt (Promega) assay. Briefly, in the dark, 20 μL of MTS was added to each well and the plate was further incubated for 1 h at 37°C, 5% CO_2_. The optical density of the resulting solution was measured at 490 nm. Results were presented as percentage of viable cells compared to the positive control. Two independent experiments, using four replicates, were performed.

#### Statistical Analysis

All graphs and statistical data analysis were performed using GraphPad Prism software package (GraphPad Software version 8.2.0). Means and standard deviation were calculated for all experimental conditions tested. Statistical analysis was carried out by ANOVA with Tukey's multiple comparison, and *p-*values < 0.05 were considered significant.

## Results

**Figure 1:**
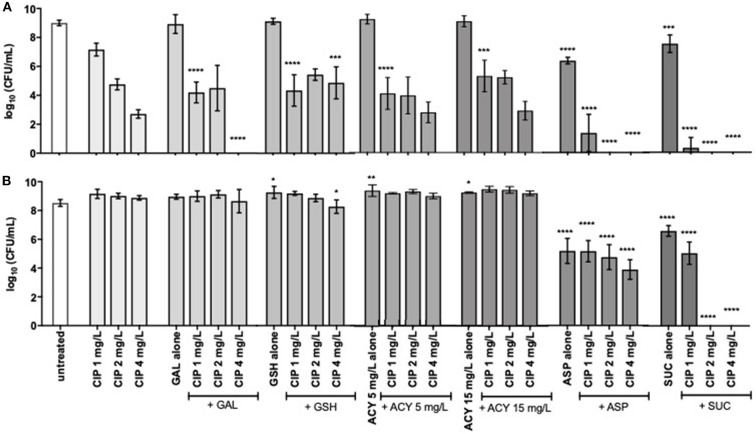
Inhibition of **(A)** *P. aeruginosa* PAO1 and **(B)** PAI growth by subinhibitory (1 and 2 mg/L) and inhibitory (4 mg/L) concentrations of ciprofloxacin alone and combined with 20 mM of galactose, aspartic acid, and succinic acid, 10 mM of glutathione, and 5 and 15 mg/L of acylases I. Values represent mean ± standard deviation of, at least, 5 independent experiments. The differences in log_10_ CFU/mL of the growth of *P. aeruginosa* strains after the application of the dual therapies were compared to ciprofloxacin when acting alone, and the differences in log_10_ CFU/mL of the growth of *P. aeruginosa* strains after the application of the compounds alone were compared to untreated populations using two-way ANOVA followed by Tukey's multiple comparison *post hoc* test. Significant differences are indicated by asterisks: *p < 0.05, **p < 0.01, ***p < 0.001, ****p < 0.0001. CFU, colony-forming units; CIP, ciprofloxacin; GAL, galactose; GSH, glutathione; ACY, acylases I; ASP, aspartic acid; SUC, succinic acid.

**Figure 2:**
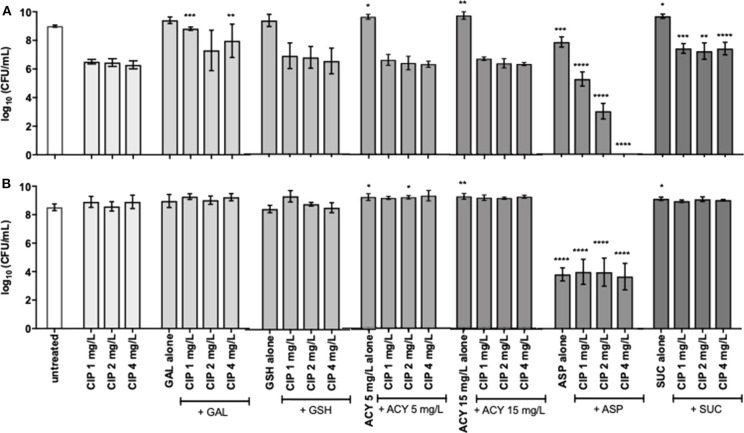
Killing activity of 24-h-old populations of **(A)** *P. aeruginosa* PAO1 and **(B)** PAI by subinhibitory (1 and 2 mg/L) and inhibitory (4 mg/L) concentrations of ciprofloxacin alone and combined with 20 mM of galactose, aspartic acid and succinic acid, 10 mM of glutathione, and 5 and 15 mg/L of acylases I against. Values represent mean ± standard deviation of, at least, 5 independent experiments. The differences in log_10_ CFU/mL of *P. aeruginosa* strains after the application of the dual therapies were compared to ciprofloxacin when acting alone, and the differences in log_10_ CFU/mL of *P. aeruginosa* strains after the application of the compounds alone were compared to untreated populations using two-way ANOVA followed by Tukey's multiple comparison *post hoc* test. Significant differences are indicated by asterisks: *p < 0.05, **p < 0.01, ***p < 0.001, ****p < 0.0001. CFU, colony-forming units; CIP, ciprofloxacin; GAL, galactose; GSH, glutathione; ACY, acylases I; ASP, aspartic acid; SUC, succinic acid.

To assess the adjuvant potential of the selected compounds, a *P. aeruginosa* strain susceptible (MIC of 0.125–0.5 mg/L) and another resistant to ciprofloxacin (MIC of 4 mg/L), PAO1 and PAI, respectively, were used on the combination studies with ciprofloxacin. The control studies of the antimicrobial activity of the compounds, when acting alone, stated that they had no antimicrobial effect with exception of aspartic acid and succinic acid (Figures 1, 2). These two compounds demonstrated some inhibitory action against the two *P. aeruginosa* strains grown in ASM. Moreover, the control studies for the action of ciprofloxacin alone were performed and the subinhibitory and inhibitory concentrations of ciprofloxacin were unable to eradicate *P. aeruginosa* grown in ASM using either a prophylactic or a treatment approach (Figures 1, 2).

### Prophylactic Application of the Dual Therapies

Results obtained for the synergistic activity of compounds and ciprofloxacin showed that, overall, prophylactic application of dual therapies showed better activity against *P. aeruginosa* than the treatment approach (Figures 1, 2). All the compounds tested enhanced the activity of, at least, one concentration of ciprofloxacin, achieving a significant bacterial load reduction of the whole *P. aeruginosa* population resident in ASM (*p* < 0.05). However, not all the compounds aided the ciprofloxacin action against both *P. aeruginosa* strains or even aided in bacterial eradication. Only aspartic acid and succinic acid demonstrated adjuvant potential against both *P. aeruginosa* strains, as evidenced by the lower number or absence of culturable cells compared to the ciprofloxacin when acting alone (Figure 1).

The improvement introduced by the aspartic acid to ciprofloxacin action was clearly evident for all concentrations tested and against both *P. aeruginosa* strains. The combination of aspartic acid with the lowest subinhibitory concentration of ciprofloxacin (1 mg/L) resulted in a significant bacterial load reduction of around 5 log for PAO1, compared with ciprofloxacin alone. This result was further improved, increasing the subinhibitory concentration of ciprofloxacin 2-fold (2 mg/L), as evidenced by the eradication of PAO1 from ASM. PAO1 eradication was also obtained combining aspartic acid with the inhibitory concentration of ciprofloxacin (4 mg/L). The dual therapy of the aspartic acid with ciprofloxacin (Asp–Cip) was more efficient against PAO1 than against the resistant strain, PAI. However, the significant load reduction of this resistant strain, ranging from 4 to 5 log, in comparison with ciprofloxacin alone must be emphasized. This reduction is notorious from a microbiological point of view.

The greatest improvements to ciprofloxacin action were introduced by succinic acid because its combination with the subinhibitory concentration of 2 mg/L of ciprofloxacin eradicated PAO1 and PAI from ASM. An identical result was obtained combining succinic acid with the inhibitory concentration of ciprofloxacin (4 mg/L).

### Application of the Dual Therapies as Treatment

As expected, the application of the dual therapies as treatment, meaning their application after 24 h of bacterial growth and biofilm formation in ASM was not as efficient as the prophylactic application. Among all the dual therapies tested, Asp–Cip was the only combination that presented anti-pseudomonal activity (Figure 2). Combined with aspartic acid, 2 mg/L of ciprofloxacin provoked a 3-log reduction of the whole PAO1 population, and 4 mg/L of ciprofloxacin seemed to eradicate the 24-h-old PAO1 planktonic and biofilm cells, in comparison with ciprofloxacin when acting alone. Aspartic acid also improved ciprofloxacin action against 24-h-old PAI populations in a similar way for the three concentrations tested, obtaining a reduction of 4 log. However, when aspartic acid acted alone, it produced identical log reduction, which led us to consider that the dual-therapy effect was caused by the action of aspartic acid and not by the combination.

### The Most Promising Dual Therapies and Their Long-Term Effect

Based on the results obtained so far, aspartic acid and succinic acid were understandably selected as the most promising adjuvants of ciprofloxacin. To select the most promising combination therapies using these compounds, two criteria were used: to provoke bacterial eradication or, at least, reduce 3 log the bacterial load present in ASM; and to have effect on the two *P. aeruginosa* strains. Based on these criteria, the Suc- and Asp-2 mg/L Cip therapies were selected for prophylactic approach and Asp-4 mg/L Cip for treatment application. Suc-2 mg/L Cip therapy eradicated both *P. aeruginosa* strains, but also Asp-2 mg/L Cip exhibited interesting results for prophylaxis since it eradicated PAO1 and significantly reduced PAI bacterial load. Asp-4 mg/L Cip was the only combination therapy applied as treatment able to eradicate the whole PAO1 population, including planktonic cells and biofilms, and reduce PAI population with microbiological significance.

**Figure 3:**
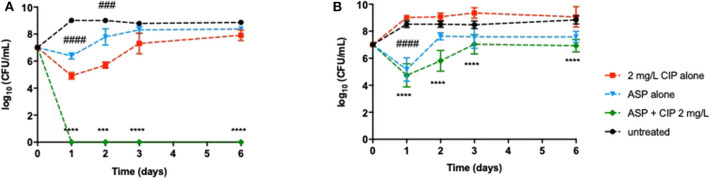
Long-term effect of the prophylactic application of 2 mg/L ciprofloxacin alone and combined with aspartic acid on **(A)** *P. aeruginosa* PAO1 and **(B)** PAI during 6 days. Values represent mean ± standard deviation of, at least, three independent experiments. The differences in log_10_ CFU/mL of *P. aeruginosa* strains after the application of Asp–Cip were compared to ciprofloxacin when acting alone (indicated by asterisks), and the differences in log_10_ CFU/mL of *P. aeruginosa* strains after the application of the aspartic acid alone were compared to untreated populations (indicated by hashtag), using two-way ANOVA followed by Tukey's multiple comparison *post hoc* test. Significant differences are indicated as follows: ***/^###^*p* < 0.001, ****/^####^*p* < 0.0001. CFU, colony-forming units; CIP, ciprofloxacin; ASP, aspartic acid.

**Figure 4:**
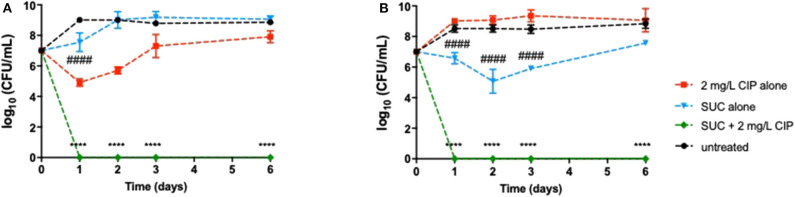
Long-term effect of the application of 2 mg/L ciprofloxacin alone and combined with succinic acid as treatment on 24-h-old populations of **(A)** *P. aeruginosa* PAO1 and **(B)** PAI during 6 days. Values represent mean ± standard deviation of, at least, three independent experiments. The differences in log_10_ CFU/mL of *P. aeruginosa* strains after the application of Suc–Cip were compared to ciprofloxacin when acting alone (indicated by asterisks), and the differences in log_10_ CFU/mL of *P. aeruginosa* strains after the application of succinic acid alone were compared to untreated populations (indicated by hashtag), using two-way ANOVA followed by Tukey's multiple comparison *post hoc* test. Significant differences are indicated as ****/^####^*p* < 0.0001. CFU, colony-forming units; CIP, ciprofloxacin; SUC, succinic acid.

Despite the promising results obtained so far, it was of utmost importance to ensure that a relapse of infection did not occur after ending the effect of the dual therapies. Relapse of infection can occur through the recovery of the population that was not completely eradicated or through the presence of dormant cells, including viable but non-culturable cells (VBNC) and persister cells (not detected in solid media). Therefore, this study aimed at investigating the behavior of the *P. aeruginosa* populations after the effect of the combination therapies had ended. The detection of VBNC and persister cells is typically performed using advanced methods, e.g., flow cytometer, but growing bacteria in ASM implies the optimization of the ASM preparation, which is not concluded in our laboratory yet. Nevertheless, a simple way to gain insights about the presence of these two kinds of dormancy states within *P. aeruginosa* populations is a long-term monitoring of the bacterial growth for several days after the application of the dual therapies (Fisher et al., 2017; Ayrapetyan et al., 2018). The results revealed that dormant cells might not yet be present in *P. aeruginosa* populations as no culturable cells were detected on the solid medium along 6 days after application of Asp-2 mg/L Cip against PAO1 (Figure 3A) and after the application of Suc-2 mg/L Cip against PAO1 and PAI (Figure 4). However, we observed that PAI load reduction provoked by Asp-2 mg/L Cip was only kept for 24 h and a full recovery of population was attained after 3 days (Figure 3B). This finding led us to consider Suc-2 mg/L Cip as the most suitable therapy to be applied as prophylaxis.

Our results also indicated that the 24-h-old PAO1 population that encompassed biofilms might not include VBNC or persister cells since no culturable cells were detected after ending the Asp-4 mg/L Cip effect, in contrast with PAI populations that recovered their initial bacterial load (data not shown).

### Effect of Asp-Cip and Suc-Cip Therapies on Polymicrobial Communities

**Figure 5:**
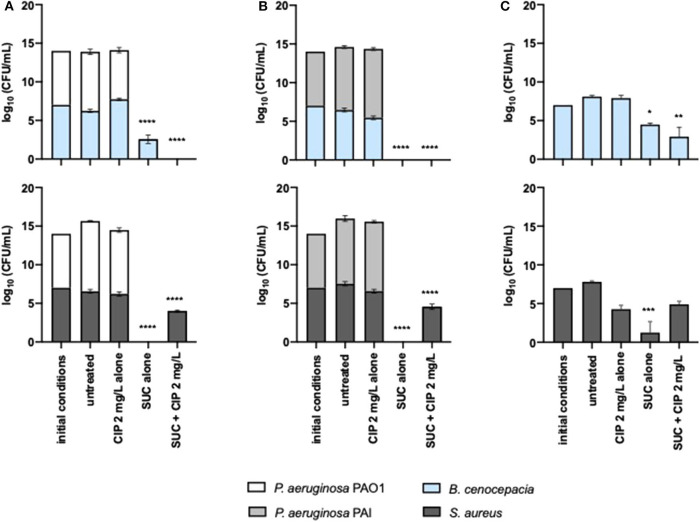
Inhibitory effect of 2 mg/L of ciprofloxacin alone and combined with succinic acid against **(A)** *P. aeruginosa* PAO1 and **(B)** PAI with *S. aureus* and *B. cenocepacia* and **(C)** against monocultures of *S. aureus* and *B. cenocepacia*. Values represent mean ± standard deviation of two independent experiments. The differences in log_10_ CFU/mL of the bacterial strains after the application of the dual therapies were compared to ciprofloxacin when acting alone, and the differences in log_10_ CFU/mL of the bacterial strains after the application of the compounds alone were compared to untreated populations using two-way ANOVA followed by Tukey's multiple comparison *post hoc* test. Significant differences are indicated by asterisks: *p < 0.05, **p < 0.01, ***p < 0.001, ****p < 0.0001. CFU, colony-forming units; CIP, ciprofloxacin; SUC, succinic acid.

We investigated the outcome of the most effective dual therapies on *P. aeruginosa* when it lives in polymicrobial communities. The adjuvant effect of succinic acid on ciprofloxacin against *P. aeruginosa* was preserved in the presence of *S. aureus* and *B. cenocepacia*, when this dual therapy was applied prophylactically (Figures 5A,B). PAO1 and PAI seemed to be identically eradicated from ASM compared to the monocultures (Figure 1). Nevertheless, we must pay much attention to the effect of the dual therapies on off-target species including *S. aureus* and *B. cenocepacia* that share a CF airway environment with *P. aeruginosa*. Suc–Cip therapy showed a positive “collateral” effect because eradicated *B. cenocepacia* along with *P. aeruginosa*. Succinic acid seemed to improve the ciprofloxacin action since this antibiotic alone was not able to eradicate this bacterial species. The interspecies interaction established between *B. cenocepacia* and *P. aeruginosa* might play a role in *B. cenocepacia* eradication because the dual therapy did not eliminate *B. cenocepacia* grown alone (Figure 5C).

In contrast to *B. cenocepacia*, the Suc–Cip therapy did not have activity on *S. aureus* growing with or without *P. aeruginosa*. Nevertheless, we noted that succinic acid alone had a distinct effect on *P. aeruginosa* and *S. aureus* compared to monocultures. This compound seemed to induce cell death of both bacterial species, whose eradication was not achieved by the Suc–Cip therapy (Figures 5A,B). In this case, ciprofloxacin seemed to compromise the action of succinic acid in the eradication of this mixed community.

**Figure 6:**
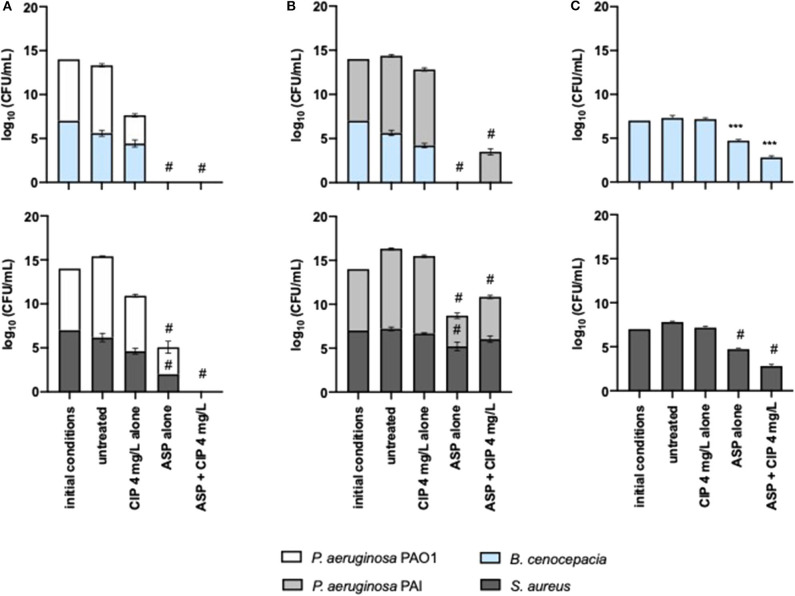
Killing effect of 4 mg/L of ciprofloxacin alone and combined with aspartic acid against mixed 24-h-old populations of **(A)** *P. aeruginosa* PAO1 and **(B)** PAI with *S. aureus* and *B. cenocepacia* and **(C)** against monocultures of *S. aureus* and *B. cenocepacia*. Values represent mean ± standard deviation of two independent experiments. The differences in log_10_ CFU/mL of the bacterial strains after the application of the dual therapies were compared to ciprofloxacin when acting alone, and the differences in log_10_ CFU/mL of the bacterial strains after the application of the compounds alone were compared to untreated populations using two-way ANOVA followed by Tukey's multiple comparison *post hoc* test. Significant differences are indicated as follows: ***p < 0.001, ^#^*p* < 0.0001. CFU, colony-forming units; CIP, ciprofloxacin; ASP, aspartic acid.

The anti-pseudomonal effect of Asp–Cip was not affected by the presence of *S. aureus* and *B. cenocepacia* (Figures 6A,B). PAO1 and PAI were identically eradicated and reduced, respectively, within 24-h-old mixed populations in comparison with the pseudomonal monocultures (Figure 2). Similar to Suc–Cip, Asp–Cip therapy demonstrated to be an interesting therapy against *B. cenocepacia* as this species was eradicated from ASM, demonstrating that aspartic acid has also functioned as adjuvant of ciprofloxacin in *B. cenocepacia* eradication. Again, interaction with *P. aeruginosa* seemed somehow to sensitize *B. cenocepacia* to eradication by Asp–Cip because monocultures of *B. cenocepacia* were not sensitive to the combination therapy (Figure 6C).

The effect of Asp–Cip on *S. aureus* within mixed 24-h-old populations was variable. When *S. aureus* shared ASM with PAO1, a sensitive strain to ciprofloxacin, Asp–Cip eradicated *S. aureus* along with *P. aeruginosa*, but when it shared with PAI, a ciprofloxacin-resistant strain, *S. aureus* survived this therapy together with PAI.

### Effect of the Most Promising Dual Therapies on the Metabolic Activity of Lung Epithelial Cells

**Figure 7:**
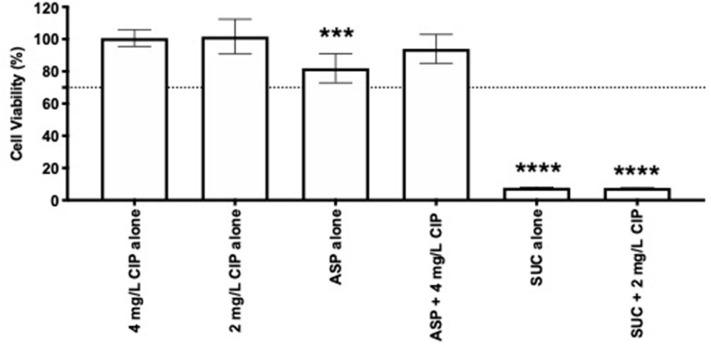
Cytotoxicity of 2 and 4 mg/L of ciprofloxacin alone and combined with succinic acid and aspartic acid, respectively. Lung epithelial cell viability was measured using MTS assay. Values represent mean ± standard deviation of two independent experiments. The viability percentage shown is compared to untreated samples. The differences in cell viability percentage compared to ciprofloxacin when acting alone were determined using one-way ANOVA followed by Tukey's multiple comparison *post hoc* test. Significant differences are indicated by asterisks: ***p < 0.001, ****p < 0.0001. CIP, ciprofloxacin; ASP, aspartic acid; SUC, succinic acid.

In addition to the anti-pseudomonal effect of the dual therapies tested, it was important to understand their effect on the human cells. Therefore, a cytotoxic assay was performed using lung epithelial cells. Ciprofloxacin and aspartic acid, alone or combined, proved to be non-toxic, as evidenced by the cell viability higher than 70% (Figure 7). Addition of succinic acid to ciprofloxacin, on the other hand, impaired the growth of these cells as demonstrated by the absence of metabolic activity. The acidic nature provided by the presence of succinic acid (Table S1), when added to the culture media, may be the reason for these results since succinic acid alone obtained identical cytotoxicity.

## Discussion

The strategies used by bacteria to resist to antibiotics are not always dependent on intrinsic features or acquiring genetic elements, but instead, resistance can be expressed through an alteration in bacterial lifestyle (Lebeaux et al., 2014b). The biofilm lifestyle is considered one of the most important adaptive mechanisms of *P. aeruginosa* within CF sputum (Høiby et al., 2010). The hallmark of biofilms is their impressive multifactorial way to resist to antibiotics that conventional treatments rarely have effect (Borriello et al., 2004; Rose and Poppens, 2009; Soares et al., 2019). Compounds that interfere with biofilm lifecycle, e.g., inhibition of the adhesion, or interfere with biofilm lifestyle, e.g., induce dispersion and disruption of matrix, are highly convenient, but most of the biofilm-controlling compounds do not kill the biofilm cells and, thus, the combination with antibiotics can be a promising strategy. Moreover, biofilm disassembly either by disruption or by dispersal is associated with a massive release of bacteria, which can represent a serious risk to patients if it would be not combined with the use of antibiotics; for instance, bacteria can enter into the bloodstream leading to sepsis (O'Toole, 2004).

Several effective anti-biofilm strategies were proposed, but until now they have not passed through clinical trials and entered the commercial market (Romero and Kolter, 2011; Lu et al., 2019). The lack of anti-biofilm agents available for the treatment of CF patients led us to rethink the choice of the biofilm-controlling compounds to be tested. Typically, researchers look for novel antibiotic adjuvants species-specific with a specific mode of action, for instance the inhibition of resistance enzymes, efflux pumps, or quorum sensing. This approach implies a great domain of species intrinsic and adaptive resistance mechanisms, including biofilm physiology and structure and the gene expression profile while forming and living in biofilms. In this study, we opted mostly for potential broad-spectrum compounds that had an extensive effect on biofilms. This kind of approach is being followed by some authors, for instance, Reffuveille et al. (2014) which reported that the synthetic peptide 1018 had broad-spectrum activity as adjuvant for different antibiotic against biofilms formed by multidrug-resistant bacteria. However, compounds such as peptide 1018 will certainly find constrains in its therapeutic approval because it is a new drug. To overcome the prolonged development timelines of therapy approval, we mainly selected FDA-approved compounds or nutrients to facilitate, in the future, the advance of the therapies for the next clinical stages.

Fluoroquinoles, in particular ciprofloxacin, is an important component of anti-pseudomonal chemotherapy in the treatment of the polymicrobial CF-associated infections (Langan et al., 2015; Emiralioglu et al., 2016), and, for this reason, it was the antibiotic chosen to search for adjuvants. Moreover, ciprofloxacin is among the priority antibiotics for which adjuvants are demanded (Brown, 2015) and our results clearly stated this need because subinhibitory and inhibitory concentrations of ciprofloxacin were unable to eradicate *P. aeruginosa* grown in ASM. It should be noted that in our study the inhibitory concentration of ciprofloxacin did not inhibit the growth of the susceptible strain. This result evidenced the difficulty that the CF airway environment poses to drug development. The presence of thick and viscous sputum in the CF lungs provides a narrow mesh that slows down or even impairs the efficient penetration of the antimicrobial molecules, resulting in the therapy failure and bacterial survival (Suk et al., 2009).

We found that aspartic acid and succinic acid revitalized ciprofloxacin action against *P. aeruginosa*. To the best of our knowledge, aspartic acid and succinic acid are not used as antimicrobial agents, for adjuvant therapy, or for other therapeutic purposes, but they are only taken as nutritional supplements (information retrieved from DrugBank database). Nonetheless, some studies have used them as biofilm-controlling compounds (Sauer et al., 2004; Parks et al., 2009; Yang et al., 2015; Bahamondez-Canas and Smyth, 2018). According to the literature findings, aspartic acid acts as an anti-adhesion and disrupting agent of biofilms formed in the CF airway environment. Parks et al. (2009) demonstrated that aspartic acid had the capacity to dissolve DNA and F-actin (released from necrotic neutrophils) bundles associated with histones. This dissociation dismantles early biofilm structures or impair biofilm formation. Even though we did not measure biofilm formation and disassembly, due to technical difficulties imposed by the ASM characteristics to separate free-floating biofilm from planktonic cells, we hypothesized that aspartic acid similarly inhibited or impaired biofilm formation, when applied prophylactically, and dismantled biofilms, when applied as treatment, as described by Parks et al. (2009). However, other mechanisms of action not related to biofilm growth should be considered. This compound when acting alone induced planktonic cell death, reducing significantly the bacterial load present in ASM, which could also contribute to the bacterial reduction or eradication of ciprofloxacin when acted along with aspartic acid.

Several studies have demonstrated that succinic acid reduces *P. aeruginosa* biofilm survival acting mainly as a dispersion agent (Sommerfeld Ross and Fiegel, 2012; Bahamondez-Canas and Smyth, 2018). However, our results of the whole biomass depletion after the application of Suc–Cip therapy showed that the adjuvant effect of succinic acid was more pronounced before bacterial growth and biofilm formation than against the 24-h-old *P. aeruginosa* populations that included biofilms, as demonstrated by Sriramulu et al. (2005). This finding could lead us to consider succinic acid for anti-biofilm (or aggregate) formation, but it would not explain the ability of ciprofloxacin to eliminate resistant bacteria. Even if ciprofloxacin-resistant bacteria, in our study represented by PAI, were unable to form biofilms or small aggregates and remained in planktonic state, resistance is still expressed, and ciprofloxacin action would be comprised. However, Suc–Cip therapy eliminated planktonic resistant bacteria, and thus we hypothesized that succinic acid somehow blocked or reduced the expression of ciprofloxacin resistance. As far as we know, this is the first report showing a different helping role of succinic acid. This synergistic action between succinic acid and ciprofloxacin must be urgently addressed because it could represent a significant advance in CF therapeutics.

A fundamental feature when an innovative therapy is under development is the ability to avoid or, at least, minimize the chances of a relapse. Our data emphasized that eradication of bacteria must be efficiently performed, otherwise bacteria can recover giving rise to a relapse of infection. Although Asp–Cip and Suc–Cip therapies have eradicated bacteria from ASM, relapse of infection can still occur due to the existence of VBNC or persister cells within *P. aeruginosa* populations. VBNC are a subpopulation of bacteria that become non-culturable on media but functionally viable and able to return to a metabolically active and culturable state (Oliver, 2010; Li et al., 2014). In turn, persister cells represent about 1% of the population that survive to antibiotic treatments despite not being antibiotic resistant (Conlon et al., 2015; Fisher et al., 2017). Both VBNC and persister cells can be induced by stressful conditions, such as an antibiotic treatment (Kubistova et al., 2017; Ayrapetyan et al., 2018) and biofilms (Pasquaroli et al., 2013; Ayrapetyan et al., 2018; Soares et al., 2019). The detection of dormant cells should be preferentially performed using advanced techniques, such as flow cytometry (Ruger et al., 2014), but the high viscosity of ASM and its components (e.g., eDNA, mucin, salts) demands an extensive optimization of the ASM preparation (before flow cytometry analysis) that we have not yet concluded in our laboratory. Therefore, we performed a long-term monitoring of bacterial regrowth as previously described (Fisher et al., 2017; Ayrapetyan et al., 2018) to gain insights about the presence of VBNC and persister cells within *P. aeruginosa* populations. The eradication effect of Asp–Cip and Suc–Cip was lasting, indicating the absence of VBNC and/or persisters within *P. aeruginosa* populations including within biofilms. Nevertheless, additional analyses should be performed to ensure the absence of VBNC and persister cells.

Asp–Cip and Suc–Cip therapies accomplished two important features of an antimicrobial CF therapy. Firstly, they exhibited anti-pseudomonal activity, and secondly, this activity had a long-term effect without bacterial regrowth. However, *P. aeruginosa* does not live alone in CF lungs. CF airway environment is a “rich soup” of microorganisms where interspecies interactions including interdependencies, competition, antagonism, and synergism among the resident members can define the survival of the *P. aeruginosa* and the infection course (Harrison, 2007; Tavernier et al., 2017). The interspecies interaction between *P. aeruginosa* and *S. aureus* is the most studied relationship in the CF context. *S. aureus* is the main responsible for the bacterial infections in younger CF patients, and it is typically a “precursor” of *P. aeruginosa* in CF lung colonization (Cystic Fibrosis Foundation, 2017). Several studies have reported that *S. aureus* can protect *P. aeruginosa* from uptake by the host immune cells and antibiotic action, promoting, for instance, the selection of SCV (Michelsen et al., 2014; Armbruster et al., 2016; Beaudoin et al., 2017). *Burkholderia* sp. predominates in older CF patients, and the relevance of the *B. cenocepacia* and *P. aeruginosa* interactions arise from some studies that reported augmented virulence potential on *P. aeruginosa*, e.g., redox-active phenazine, peroxides, rhamnolipids, hydrogen cyanide, and the siderophore pyoverdine (Tomlin et al., 2001; Bakkal et al., 2010; Bragonzi et al., 2012; Costello et al., 2014; Tavernier et al., 2017). These phenotypic characteristics are quite found in CF isolates of *P. aeruginosa* and fundamental for the success of infection establishment (Sousa and Pereira, 2014). Despite competing for the same resources, including oxygen and nutrients, *P. aeruginosa* and *Burkholderia* sp. are able to coexist in biofilms, triggering an increased inflammatory response (Riedel et al., 2001; Bragonzi et al., 2012). According to our results, *S. aureus* and *B. cenocepacia* did not affect the anti-pseudomonal activity of Asp–Cip and Suc–Cip therapies, which reinforced their potential as novel CF therapeutic option.

Broad-spectrum antibiotic adjuvants can bring about serious side effects on CF microbiome. It is not desirable that the eradication of *P. aeruginosa* from the CF airway environment provides a window of opportunity for other bacterial species to thrive. Moreover, at this point, Asp–Cip and Suc–Cip therapies have had an impressive performance. The eradication of *P. aeruginosa* from the polymicrobial communities did not favor the growth of *S. aureus* or *B. cenocepacia*. Indeed, aspartic acid and succinic acid also act as ciprofloxacin adjuvants against *B. cenocepacia*.

In order to appreciate the feasibility of the Asp–Cip and Suc–Cip therapies for CF application, their cytotoxicity was evaluated. The choice of nutrients as ciprofloxacin adjuvants showed some benefits, because Asp–Cip therapy was not toxic to lung epithelial cells. Although being a nutrient as well, succinic acid was toxic, which conferred toxicity to Suc–Cip therapy. This might result from the acidity of succinic acid damaging the lung epithelial cells. It would be interesting to investigate if succinic acid preserved its adjuvant potential at lower and non-cytotoxicity concentrations.

In conclusion, we evidenced that the combination of an antibiotic with adjuvants with biofilm-controlling features could be the next wave of antimicrobial CF therapeutics to combat polymicrobial CF-associated infections. We used ASM containing the major components of CF sputum, which yielded *P. aeruginosa* populations, including planktonic cells and free-floating biofilms, much more identical to the *in vivo* airway CF conditions. We found that succinic acid and aspartic acid, two nutrients, were promising ciprofloxacin adjuvants in the inhibition and eradication of bacterial growth and biofilm formation. Succinic acid and aspartic acid could allow the lifespan of ciprofloxacin to be extended in CF therapeutics as well as in other disease contexts. Despite their evident potential, more studies must be performed in order to tune the adjuvant concentration and determine the synergistic mechanism of action underlying these combinations.

## Data Availability Statement

The datasets generated for this study are available on request to the corresponding author.

## Author Contributions

All authors listed have made a substantial, direct and intellectual contribution to the work, and approved it for publication.

## Conflict of Interest

The authors declare that the research was conducted in the absence of any commercial or financial relationships that could be construed as a potential conflict of interest.
