# Supplementary material for: Fostering Innovation in the Treatment of Chronic Polymicrobial Cystic Fibrosis-Associated Infections Exploring Aspartic Acid and Succinic Acid as Ciprofloxacin Adjuvants
Source: Front Cell Infect Microbiol. 2020 Aug 27;10:441. doi: 10.3389/fcimb.2020.00441 (PMC7481398; doi:10.3389/fcimb.2020.00441)
Supplement: Supplementary file 1 [file Table_1.pdf]

Table S1. pH measurements of ASM after the application of the mono and dual therapies. Values represent mean ± standard deviation of 5 measures.

**ASM before sterilisation**	7.01 ± 0.01
**ASM after sterilisation**	6.82 ± 0.02

	**24 h**	**48 h**
**ASM (37º C)**	6.78 ± 0.02	6.75 ± 0.01

**Time after application**	**20 mM SUC**	**2 mg/L CIP**	**SUC-CIP**	**20 mM ASP**	**4 mg/L CIP**	**ASP-CIP**
**0 h**	3.99 ± 0.02	6.88 ± 0.01	3.99 ± 0.03	10.07 ± 0.03	6.90 ± 0,02	10.07 ± 0.02
**24 h**	3.98 ± 0.02	6.79 ± 0.02	4.00 ± 0.01	9.38 ± 0.01	6.99 ± 0.02	9.47 ± 0.02

ASP – aspartic acid; CIP – ciprofloxacin; SUC – succinic acid.
